# Dynamic Information Flow Based on EEG and Diffusion MRI in Stroke: A Proof-of-Principle Study

**DOI:** 10.3389/fncir.2018.00079

**Published:** 2018-10-01

**Authors:** Olena G. Filatova, Yuan Yang, Julius P. A. Dewald, Runfeng Tian, Pablo Maceira-Elvira, Yusuke Takeda, Gert Kwakkel, Okito Yamashita, Frans C. T. van der Helm

**Affiliations:** ^1^Department of Biomechanical Engineering, Delft University of Technology, Delft, Netherlands; ^2^Department of Physical Therapy and Human Movement Sciences, Feinberg School of Medicine, Northwestern University, Chicago, IL, United States; ^3^Clinical Neuroengineering, Centre for Neuroprosthetics, Swiss Federal Institute of Technology (EPFL), Clinique Romande de Réadaptation, Sion, Switzerland; ^4^Center for Advanced Intelligence Project, RIKEN, Tokyo, Japan; ^5^Neural Information Analysis Laboratories, ATR, Kyoto, Japan; ^6^Department of Rehabilitation Medicine, Amsterdam Neurosciences and Amsterdam Movement Sciences, University Medical Centre Amsterdam, Amsterdam, Netherlands

**Keywords:** EEG, diffusion MRI, somatosensory evoked potentials (SEP), brain dynamics, stroke

## Abstract

In hemiparetic stroke, functional recovery of paretic limb may occur with the reorganization of neural networks in the brain. Neuroimaging techniques, such as magnetic resonance imaging (MRI), have a high spatial resolution which can be used to reveal anatomical changes in the brain following a stroke. However, low temporal resolution of MRI provides less insight of dynamic changes of brain activity. In contrast, electro-neurophysiological techniques, such as electroencephalography (EEG), have an excellent temporal resolution to measure such transient events, however are hindered by its low spatial resolution. This proof-of-principle study assessed a novel multimodal brain imaging technique namely Variational Bayesian Multimodal Encephalography (VBMEG), which aims to improve the spatial resolution of EEG for tracking the information flow inside the brain and its changes following a stroke. The limitations of EEG are complemented by constraints derived from anatomical MRI and diffusion weighted imaging (DWI). EEG data were acquired from individuals suffering from a stroke as well as able-bodied participants while electrical stimuli were delivered sequentially at their index finger in the left and right hand, respectively. The locations of active sources related to this stimulus were precisely identified, resulting in high Variance Accounted For (VAF above 80%). An accurate estimation of dynamic information flow between sources was achieved in this study, showing a high VAF (above 90%) in the cross-validation test. The estimated dynamic information flow was compared between chronic hemiparetic stroke and able-bodied individuals. The results demonstrate the feasibility of VBMEG method in revealing the changes of information flow in the brain after stroke. This study verified the VBMEG method as an advanced computational approach to track the dynamic information flow in the brain following a stroke. This may lead to the development of a quantitative tool for monitoring functional changes of the cortical neural networks after a unilateral brain injury and therefore facilitate the research into, and the practice of stroke rehabilitation.

## Introduction

Stroke is a sudden interruption of the blood supply to the brain due to a vessel occlusion or rupture (World Health Organization, 2015). After the incident, most survivors suffer from hemiparesis, making it more difficult to perform activities of daily living. Clinical tests, such as Fugl-Meyer motor scores, indicate the severity of neural impairment following a stroke, but do not provide insight to the changes within the brain that occur after the incident and during recovery (Gladstone et al., 2002). Brain plasticity or neuroplasticity refers to the ability of the brain to reorganize neuronal connections, triggered by goal-oriented and environment-induced experiences—thus learning and adapting (Arya et al., 2011). The problem of understanding how the brain reconfigures itself following a stroke may be approached in different ways. One of the main strategies is to investigate brain responses to external stimuli. This can be achieved with various non-invasive brain imaging techniques such as electroencephalography (EEG) and functional magnetic resonance imaging (fMRI) (Bandara et al., 2016; Weinstein et al., 2017).

Mapping from the measured scalp EEG signals to their cortical sources is called an inverse problem, which is inherently ill-posed due to a limited number of measurement electrodes in comparison to the large number of active sources in the cortex (Wendel et al., 2009). Thus, precise source localization is a key challenge for EEG. Despite its poor spatial resolution, the major advantage of EEG is its temporal resolution in the order of milliseconds, which allows capturing fast dynamics of neuronal activity in the brain. A recent review of stroke rehabilitation indicated that the assessment of electrical neuronal activity with EEG may provide a precise way of measuring dynamic neural processes and thereby providing biomarkers for time-dependent brain plasticity during spontaneous neurobiological recovery (Ward, 2017). In contrast, the spatial resolution of fMRI is in the order of 2–3 mm, which is much higher than that of EEG. However, the temporal resolution of fMRI is relatively low because the hemodynamic response reaches its peak around 5–6 s after the neural activity. Moreover, the fMRI is an indirect measure of electrical neuronal activity in the brain (Heeger et al., 2000).

Additional to the functional brain imaging approaches, anatomical brain imaging techniques are also often used in the stroke research (Qiu et al., 2011; Song et al., 2012, 2015; Wirsich et al., 2017). For example, commonly used T_1_-weighted structural MRI allows obtaining the high-resolution detailed brain structure. Diffusion-weighted MRI (dMRI) is another anatomical acquisition that is typically used to infer white matter connections between cortical regions (Owen et al., 2017). Anatomical brain imaging techniques reflect the anatomical changes in the brain following a stroke; however, they cannot provide direct insight into functional changes brain activity caused by a brain lesion (Boyd et al., 2017).

As discussed, each brain imaging technique has its pros and cons (Ward, 2015; Boyd et al., 2017). Nowadays, it has become clear that combining different imaging modalities may improve our understanding of the brain as a complex biological system and its functions (Arikan, 2011). The excellent temporal resolution of EEG provides unique advantage for monitoring dynamic changes of neuronal activity at thecortex following a stroke. Nevertheless, the underdetermined nature of the inverse problem of EEG calls for structural, physiological and functional information to be combined to better estimate the location of active sources at the cortex and the causal interactions between sources, i.e., effective connectivity, related to a specific form of stimulus. Various computational approaches such as dynamic causal modeling (DCM) and conditional Granger causality analysis have been proposed and used to estimate the effective connectivity (Bajaj et al., 2015; Schulz et al., 2016; Wang et al., 2016). However, most of the current methods either require prior assumptions on the model structure (e.g., DCM) or exclusively rely on the signal correlations without considering anatomical constraints in the model (e.g., Granger causality analysis). Among the state-of-the-art methods, the Variational Bayesian Multimodal Encephalography (VBMEG) method has shown potential both in locating the active cortical sources and in identifying neural pathways (both physically and causally) between them, without involving prior assumptions on the model structure. A physiologically constrained Bayesian estimation algorithm is used to locate active cortical sources. Combining them with white matter tracks estimated from dMRI, a linear connectome dynamics (LCD) model is built to infer causal interactions between active cortical sources (Friston, 2011). Such a method allows tracking the information flow through the neural fibers within the brain network. The VBMEG method was initially proposed to investigate the dynamic cortical activity of healthy participants during a face recognition task (Fukushima et al., 2015). VBMEG has been tested in both simulations (Sato et al., 2004; Aihara et al., 2012) and healthy volunteer studies (Yoshioka et al., 2008; Yoshimura et al., 2012, 2017; Nakamura et al., 2015); their main focus was on muscle activity reconstruction and visual stimuli analysis with or without structural and functional MRI constraints. Nevertheless, as a novel brain imaging method, the clinical value of the VBMEG method is yet to be demonstrated regarding its potential to investigate functional brain changes following a brain disease such as a stroke.

Therefore, the present work serves as a proof-of-principle study demonstrating the feasibility of the VBMEG method to estimate active cortical sources and their dynamic interactions in stroke participants during a sensory stimulation task. The high-density EEG, anatomic MRI, and diffusion MRI data were collected from both able-bodied and stroke participants. EEG was recorded when the participants were receiving electrical finger stimulation. The accuracy of EEG source localization and dynamic information flow estimation within the VBMEG method was evaluated by the Variance Accounted For (VAF). The VAF indicates how much cortical activity and brain dynamics can be explained by the VBMEG method. The estimated dynamic information flow was compared between two chronic hemiparetic stroke survivors and two able-bodied individuals to demonstrate the feasibility of the VBMEG method in revealing functional cortical network changes post-hemiparetic stroke. This proof-of-principle study is a critical prerequisite for applying the VBMEG on a large database to identify a quantitative biomarker for assessing neurological impairment and exploring neurobiological recovery following a stroke.

## Materials and methods

### Subjects

**Figure 1:**
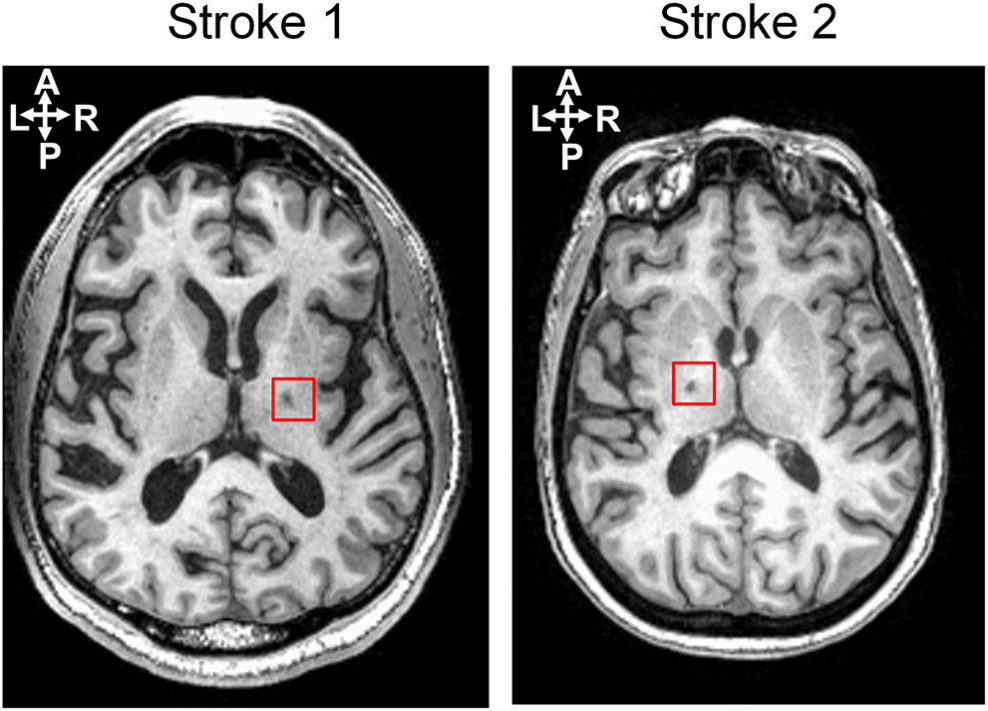
Lesion locations for two stroke subjects shown on an axial slice of the T1-weighted anatomic images.

Two chronic stroke survivors and two age-matched able-bodied individuals were included in this proof-of-principle study. The participants were recruited with informed consent and permission of the Medical Ethics Committee of the Vrije Universiteit Medical Center, Amsterdam. The trial protocol was registered on 23 October 2013 at the Netherlands Trial Register (identifier NTR4221). Inclusion criteria for the subjects suffering from chronic stroke were (1) upper limb paresis, (2) ability to sit without support (National Institutes of Health Stroke Scale item 5a/b > 0), (3) age over 18, (4) single ischemic hemispheric stroke, (5) more than 6 months post-stroke. Exclusion criteria were (1) previously existing pathological neurological conditions or orthopedic limitations of the upper limb that would affect the results, (2) botulin toxin injections or medication that may have influenced upper limb function in the past 3 months, (3) general MRI contraindications (claustrophobia, pacemaker, or other metallic implants), and (4) absence of history of epilepsy or seizures. All participants are in the age range of 55–70 in this study. The information of lesion side in the brain and clinical assessment for stroke survivors is provided in the Table 1. Both stroke participants had lesions in the posterior limb of internal capsule, but in different hemispheres, as shown in Figure 1.

**Table 1:** Information of stroke subjects.

**Subject**	**Lesion side**	**FM-UE**	**EmNSA**	**Year of stroke**
Stroke 1	Right	58	8	2009
Stroke 2	Left	66	8	2009

*FM, Fugl-Meyer Upper Extremity Assessment Score; EmNSA, the Erasmus MC modification of the Nottingham Sensory Assessment*.

The finger stimulation experiment and EEG data acquisition were performed in a conversion van which was designed to execute measurements at geographical locations convenient for the participants. MR images were acquired on another day after the EEG recording was completed at VU University Medical Center at Amsterdam. The two stroke participants were chronic and the above measurements were done more than 6 years post-stroke, meaning that their recovery had plateaued.

### Electrical finger stimulation and EEG acquisition

The experiment was performed within a NEN1010 approved measurement van. During the experiment, participants were sitting comfortably with their hands and forearms positioned on their lap with the fingers facing upward (supine position). Between forearm and lap, a pillow was placed to secure a stable position and comfort. Index fingers of both hands were stimulated with a randomized order in healthy controls and stroke patients with bipolar stimulation using a battery-powered electrical stimulator (Micromed, Brain quick, Treviso, Italy). The anodal electrode (size 1 cm) was placed on most distal phalange and cathode on the second distal phalange with an inter-electrode distance of ~1 cm (Kalogianni et al., 2018a). This placement is chosen to reduce the likelihood of anodal block (Cruccu et al., 2008). A monophasic anodic rectangular electrical pulse of 400 μs width and a stimulation intensity of two times the sensation threshold was chosen. The sensation threshold was defined as the level at which the subject was able to sense half of the 10 given pulses (Jones and Tan, 2013). The chosen stimulation did not cause any pain or heat feeling to the participants.

The finger stimulation was repeated during 500 trials for each hand. During the stimulation, the EEG data were recorded with a 64-channel EEG system (TMSi, Netherlands) with ground electrode placed at the left mastoid, and online referenced to the common average. Sampling rate was 1,024 Hz. Apart from antialiasing filters, no other filters were applied online. Positions of the EEG electrodes for every subject were measured with the ANT Neuro Xensor system (ANT Neuro, Enschede, Netherlands). The experimental setup (e.g., EEG cap placement and preparation, etc.) and finger stimulation had a typical duration of 50 min per participant. This short experimental time is plausible for stroke participants without any physical or mental fatigue.

### EEG pre-processing

EEG data were preprocessed using EEGLAB (Delorme and Makeig, 2004), which is an open source toolbox running in the MATLAB environment. Continuous EEG data was band-pass filtered between 1 and 30 Hz to remove possible slow trends in the data (e.g., blood pressure, heartbeat, and breathing) and high-frequency fluctuations in event-related potentials, and then down-sampled to 512 Hz. EEG epochs were extracted using a window analysis time of 250 ms, with 50 ms before stimulus and 200 ms after stimulus. The artifact caused by electrical stimulus was removed by a blanking window from 10 ms before the stimulus to 10 ms after the stimulus. Then the gap was filled by a 3-order autoregressive model. Independent Component Analysis (ICA) algorithm (Delorme and Makeig, 2004) was used to remove the components of eye-blinks and movements (Li et al., 2006). After the artifact removal, the baseline correction was applied to each epoch using the signal from 50 to 10 ms before the stimulus. The epochs for the same experimental conditions were averaged in each subject, time-locked to the onset of the stimulus to extract the event-related potential (ERP).

### MRI acquisition and preprocessing

Image acquisition was performed with a 3T MRI scanner (Discovery MR750, GE Medical Systems) at VU University Medical Center. Anatomical T1-weighted acquisition had the following settings: TE = 3.22 ms, TR = 8.21 ms, flip angle 12°, imaging matrix = 256 × 256 × 172, resolution 1 mm^3^. The diffusion-weighted MRI (dMRI) acquisition protocol involved 40 non-collinear gradient directions uniformly sampled over a sphere for each of two *b*-values: 1,000 and 2,000 s/mm^2^; TE = 100 ms, TR = 7,200 ms, voxel size 2.5 × 2.5 × 2.5 mm^3^, 52 consecutive slices, acquisition time 12.5 min. This allowed for whole brain coverage. Data for each *b*-value were acquired as separate scans together with five non-diffusion weighted images (i.e., per *b*-value).

The dMRI data were preprocessed using FSL v5.0 (http://fsl.fmrib.ox.ac.uk/fsl/) (Jenkinson et al., 2012). The acquired DWIs were corrected for motion and eddy current distortion by affine co-registration to the reference b_0_-image (using FSL eddy_correct). Gradient directions were reoriented according to the rotation component of the affine transformation. Diffusion tensor fitting and fractional anisotropy (FA) were calculated using FSL, and fiber tracking was performed withMRTrix software v0.2.10 (http://jdtournier.github.io/mrtrix-0.2/index.html).

### VBMEG method

**Figure 2:**
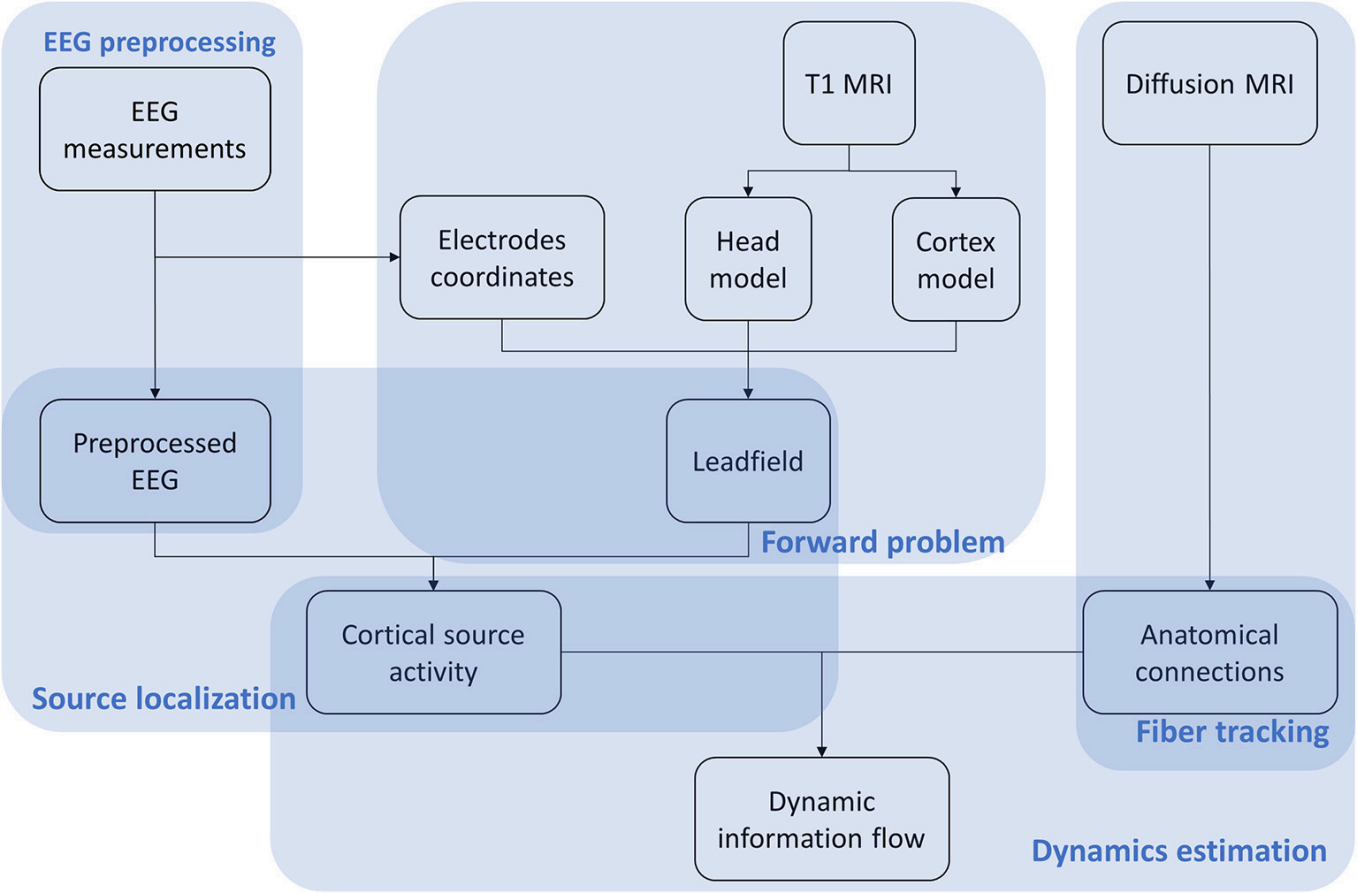
Workflow of the VBMEG method. EEG, anatomic and diffusion weighted MRIs are first preprocessed. Then EEG sources are estimated using hVB approach. By combining sources and anatomical connections extracted from diffusion MRI, the linear connectome dynamics model is built leading to the estimation of dynamic information flow traveling between sources. The results are visualized individually for each subject dataset.

The VBMEG method is constituted by a hierarchical Variational Bayesian (hVB) estimation of cortical sources as proposed by Sato et al. (2004) and a dynamic estimation of the information flow traveling from one source to another (Fukushima et al., 2015). In contrast to the original work from Sato et al. (2004), prior knowledge obtained from functional MRI was not included in hVB estimation. In this study, the source localization and dynamic information flow estimation were performed using VBMEG toolbox with default settings, using pre-processed EEG data, built leadfield matrix and fiber tracking results. The VBMEG toolbox and documentation are available online (http://vbmeg.atr.jp/docs/v2/static/vbmeg_users_manual.html, http://vbmeg.atr.jp/download2/). The general overview of the VBMEG method is provided in Figure 2.

#### Source localization

An individual head model for each subject was built using the T1 MR image for EEG source localization. Freesurfer (Reuter et al., 2012), an MRI processing software, was used to construct a polygon model of cortical surface, label the cortex surface anatomically, and extract the inner skull surface and outer scalp surface from the T1 image. Then a three-layer (CSF, skull, and scalp) head model was built using boundary element method (BEM) by VBMEG toolbox (Fukushima et al., 2015). Ten thousand vertices on the cortex surface were chosen as possible dipole sources, and the leadfield matrix was built based on the position of dipole sources and EEG electrodes, as well as the head model by VBMEG toolbox. The conductivity of CSF, skull, and scalp was set as 0.62, 0.03, and 0.62 s/m respectively as the default setting in the toolbox (ATR, 2017). As both stroke participants have small white matter lesions in the deep brain area (see Figure 1), their lesions would not affect EEG source localization.

The inverse calculation was performed using the hVB method to estimate the cortical source of EEG activity. The hVB is an altered version of the MNE method similar in structure to the Wiener filter, which intends to use the current variance to regularize the solution of the L2- reconstruction problem (Sato et al., 2004). Nevertheless, because the true current variance is unknown, the hVB method places a hierarchical prior on the current variance and estimates it iteratively using an Automatic Relevance Determination (ARD) model (Neal, 1996). The hVB method differentiates itself further from the MNE method in that it places a smoothness constraint in the currents, ensuring that neighboring active sources are correlated (Sato et al., 2004). The source activity was estimated with pre-processed EEG signals and estimated leadfield matrix using the hVB method, which is implemented in the VBMEG toolbox.

#### Dynamic information flow estimation

The dynamic information flow was estimated by a LCD model (as a variant of multivariate autoregressive, MAR model) to determine whether causal interactions exist between active cortical sources (Fukushima et al., 2015). The time window for dynamic analysis is around 0–200 ms post-stimulation for the length of 102 samples (sampling rate: 512 Hz). An anatomical constraint was applied to the LCD model based on the fiber tracking results from diffusion MRI, so only the anatomically connected sources have the non-zero weights. For fiber tracking, the cortical surface was parcellated into 250 equally distributed target regions of interest (ROIs) based on the diffusion MRI data. The remaining cortex vertices were clustered into these ROIs based on their spatial proximity. The source activity of these ROIs was calculated as the mean of contained dipole moments. Thus, there are 250 variables in the LCD model. Fibers connecting these ROIs through white matter were tracked using MRtrix 0.2. The fiber tracking results provide information about the presence of fiber connections between ROIs as well as the length of the fibers. The time lags in the LCD model were estimated based on the length of fiber connection using the theoretical conduction velocity of axon equal to 6 m/s (Fukushima et al., 2015). Only the terms with specific time lags were included in the LCD model, therefore the order for inter-variable interaction is one. In this case, the estimated LCD model could be represented by a 2-D matrix for inter-source dynamics. The intra-source dynamics was set as a second-order interaction. The LCD weights were estimated based on fiber connections and their corresponding time lag using an L_2_-regularized least-squares method with the default regularization parameter (0.01) (Golub and Reinsch, 1971). These LCD weights represented the estimated dynamic information flow between cortical sources.

### Model evaluation

The accuracy of source localization and dynamic information flow estimation was evaluated by calculating the VAF (Vlaar et al., 2017; Kalogianni et al., 2018b). For source localization, the estimated sources were used to generate an estimated EEG signal $\hat{M}=L\hat{S}$, which was compared with collected EEG signal ***M***. For the ith EEG channel, ***VAF***_***M***_***i***__ was defined as:

$$\begin{array}{l} VAF_{M_{i}}=\left(1-\frac{\mathrm{var}\left(M_{i}-{\hat{M}}_{i}\right)}{\mathrm{var}\left(M_{i}\right)}\right)\cdot 100\%. \end{array}$$

The time window was chosen as from 0 to 200 ms. As EEG channels on the non-active areas are not representative, the VAF for source localization ***VAF***_***M***_ was defined as the median (instead of mean) of ***VAF***_***M***_*i*__ across all EEG channels. For dynamic information flow estimation, one step forward (2 ms) of source activity $\hat{S}$ was estimated by the LCD model. The estimated source activity was compared with the results ***S*** from source localization. For a specific time point *t*, the ***VAF*_*S*_(*t*)**was defined as:

$$\begin{array}{l} VAF_{S}\left(t\right)=\left(1-\frac{\mathrm{var}\left(S\left(t\right)-\hat{S}\left(t\right)\right)}{\mathrm{var}\left(S\left(t\right)\right)}\right)\cdot 100\%, \end{array}$$

where ***S*(*t*)** and $\hat{S}\left(t\right)$ are vectors containing all source activities resulting from source localization and estimated from LCD model respectively, and *t* is the time going from 0 to 200 ms. The VAF for dynamic information flow ***VAF*_*S*_** was defined as the mean of ***VAF*_*S*_(*t*)** in the time window.

As the accuracy of the LCD model can be affected by the signal to noise ratio (SNR), the SNR of the EEG recording was also calculated. The SNR is defined as follows:

$$\begin{array}{l} SNR=\frac{A_{RMS_{signal}}}{A_{RMS_{noise}}}, \end{array}$$

where A_RMS_ is the root mean square amplitude. To intuitively show the signal level, signal percentage was calculated by

$$\begin{array}{l} P_{signal}=\frac{A_{RMS_{signal}}}{A_{RMS_{signal}}+A_{RMS_{noise}}}\cdot 100\% \end{array}$$

## Results

The results of the method application are illustrated in four cases: for two able-bodied individuals and two chronic stroke subjects.

**Figure 3:**
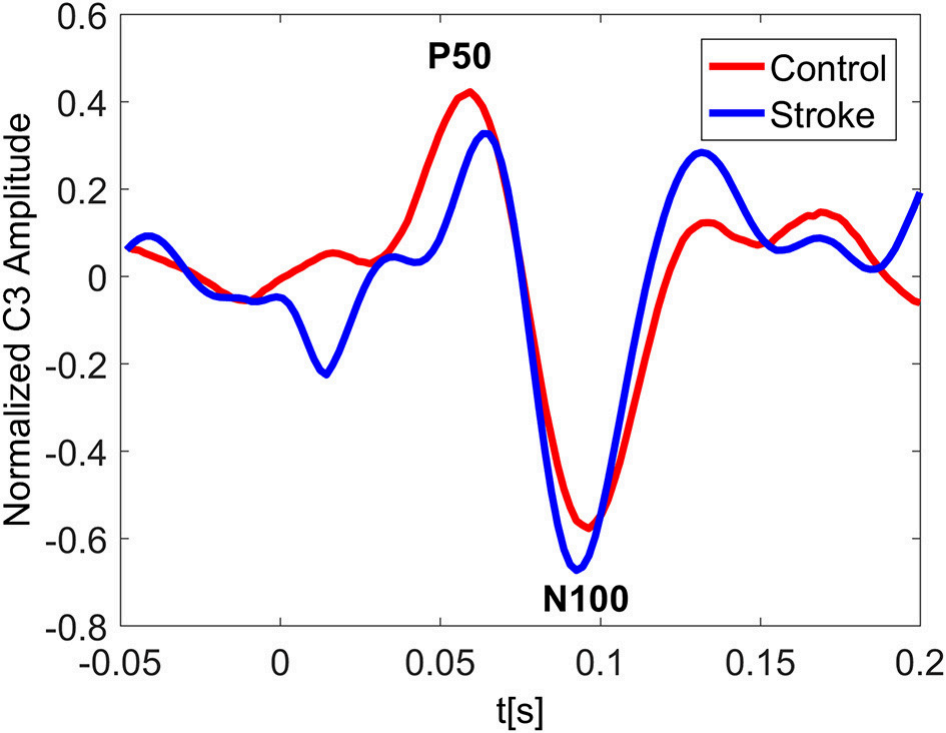
Normalized ERP at the channel C3 for a control and a stroke subject with stimulus on right hand. The ERP plotting at C3 shows great similarity for both control and stroke. The latency of P50 peak for stroke is slightly larger than that of control.

**Figure 4:**
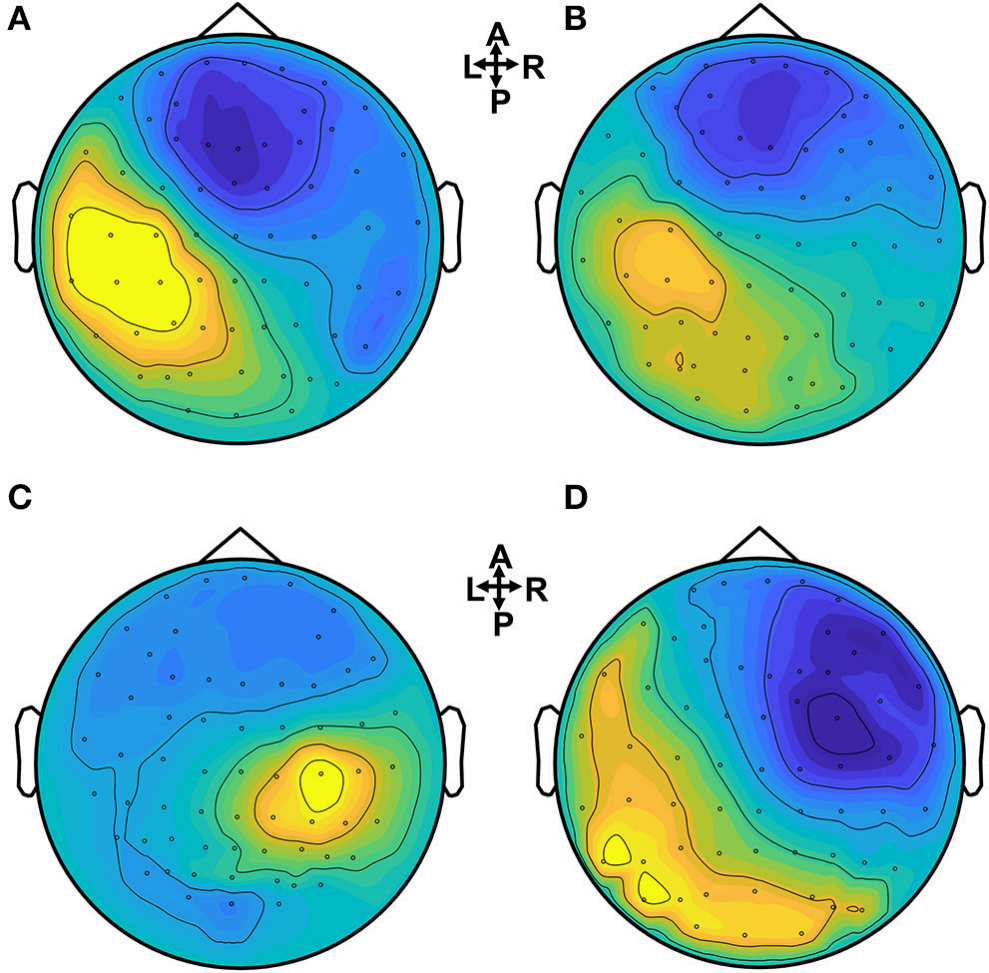
Brain topographies of the P50 peak for control and stroke subjects when a dominant hand (for controls) or an affected hand (for stroke) is stimulated. **(A,B)** Controls, **(C)** stroke 1, **(D)** stroke 2.

In Figure 3 the ERP of a control and a stroke subject are presented. In line with the literature (Oniz et al., 2016; Zhang et al., 2016), a positive-going peak around 50 ms (P50) and a negative-going peak around 100 ms (N100) were identified in the ERP for both control and stroke. Additionally, we provide the ERP topographies at the latency of P50 in Figure 4. Both controls have similar topographies with large ERP values at the sensorimotor area of the contralateral hemisphere. This result is consistent with previous studies (Desmedt and Cheron, 1980; Buchner et al., 1995; Druschky et al., 2003). Individual differences are shown in stroke patients, which may be related to subject-specific lesion load and recovery.

The VAF of EEG source localization is shown in Table 2, where we can see the VAF of source localization is higher than 80% for all subjects.

**Table 2:** The VAF of EEG source localization (inverse model) for each subject.

**Subject**	**VAF, right hand (%)**	**VAF, left hand (%)**
Control 1	94.49	96.28
Control 2	93.64	92.27
Stroke 1	90.03	87.12
Stroke 2	85.63	83.79

**Figure 5:**
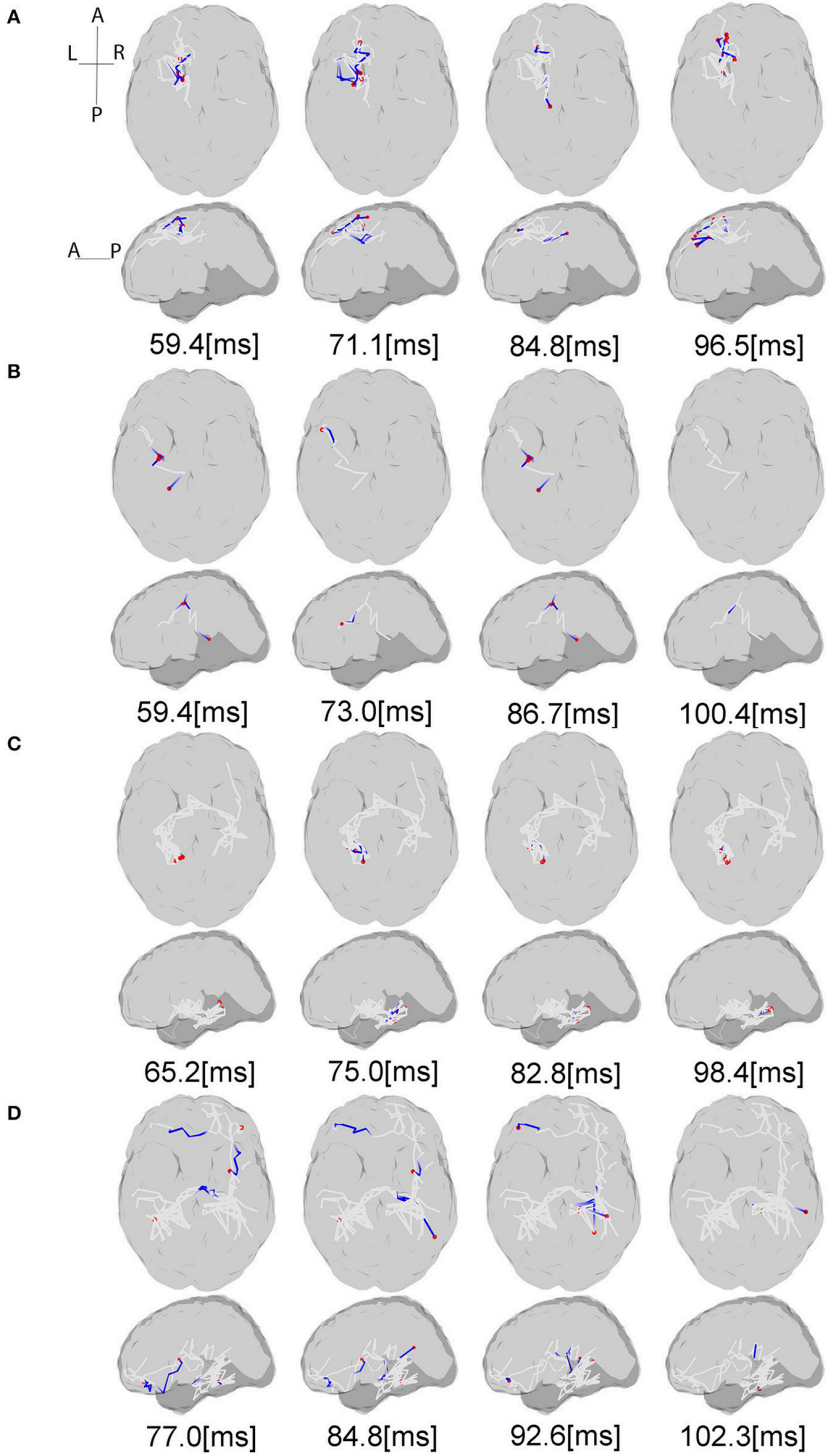
Source interactions estimated from LCD model. The plots show the information flow between P50 and N100 for each subject and anatomic connections between the active sources estimated via white matter tractography based on the individual dMRI acquisitions. The gray lines indicate the whole fiber network involved in the transmission of somatosensory information flow through the brain. The blue lines show the currently active fibers, and red dots are the currently active sources on the cortex at the specific time points. The “active sources” here denotes the sources have electrical neural activities at the presented time point, while “active fibers” indicate the fibers where the information flow is traveling through. For each subject projection of all axial slices (top) and of all sagittal slices (bottom) are shown. **(A,B)** Controls, **(C)** stroke 1, **(D)** stroke 2. For the full visualization see Supplementary Material.

Figure 5 shows the estimated dynamic information flow for each subject for finger stimulation at the dominant hand for control subjects, and at the affected hand for stroke participants. It also schematically depicts the anatomic connections between the active sources. The information flow is shown only at the contralateral hemisphere in the control subjects, while at the both hemispheres in the stroke participants. In the time period between P50 and N100 peaks, information flow occurs in the ipsilateral (contralesional) hemisphere, i.e., the left hemisphere for stroke subject 1 and the right hemisphere for stroke subject 2.

The VAF of dynamic information flow estimation is provided in Table 3, where the VAF is higher than 90% for all subjects. Additionally, we also provide the SNR for all subjects in Table 4. Although the SNR for the stroke subjects is slightly lower than the controls, the signal percentage is above 88% for all subjects. To determine the baseline value of VAF when the input signal of the model is random, we replaced ERP signals with white noise. The same estimation and prediction process were repeated 100 timed with different noise realizations to determine the baseline. The estimated VAF obtained from this baseline test was around zero. Therefore, the high VAF from our dynamic information flow estimation, with respect to EEG source activity, can prove the significance of our results by comparing it to this baseline.

**Table 3:** The average VAF of the dynamic model estimation with standard deviation for all subjects.

**Subject**	**VAF right hand**		**VAF left hand**	
	**Mean (%)**	**Std (%)**	**Mean (%)**	**Std (%)**
Control 1	97.77	12.42	97.77	12.41
Control 2	97.58	10.00	97.78	12.42
Stroke 1	92.30	14.80	93.75	12.06
Stroke 2	91.69	11.58	92.86	10.47

**Table 4:** Signal to noise ratio of data acquisition in each subject when the corresponding hand was stimulated.

**Subject**	**Right hand**		**Left hand**	
	**SNR (dB)**	**Signal (%)**	**SNR (dB)**	**Signal (%)**
Control 1	14.22	96.35	13.76	95.97
Control 2	13.45	95.68	15.28	97.12
Stroke 1	7.64	85.30	8.62	87.92
Stroke 2	9.92	90.76	8.71	88.13

*In Stroke 1 case left hand was impaired. In Stroke 2 case right hand was impaired*.

**Figure 6:**
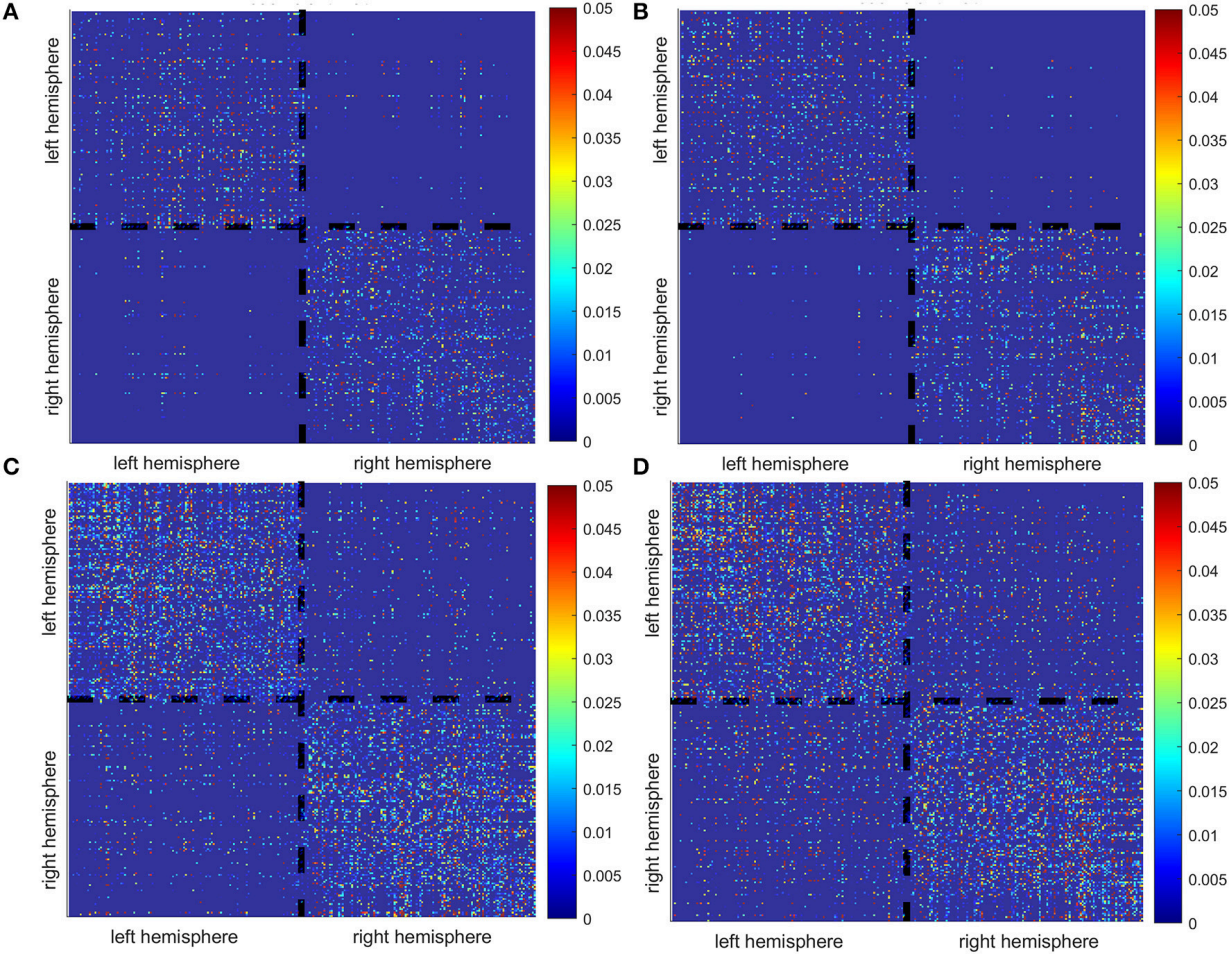
Matrices of the LCD model coefficients for controls **(A,B)** and stroke participants **(C,D)**.

For each subject, estimated coefficients matrices of the LCD model are presented in Figure 6, where we can see that increased inter-hemisphere interactions are shown for the stroke participants. This increase is also characterized by the number and percentage of the non-zero LCD model coefficients within and between hemispheres as shown in Table 5.

**Table 5:** Number and percentage of intra-hemispheric vs. inter-hemispheric interactions represented by non-zero LCD model coefficients.

	**Intra-hemispheric interactions**		**Inter-hemispheric interactions**	
	**Number of interactions**	**Percentage**	**Number of interactions**	**Percentage**
Control 1	4,956	89.3	594	10.7
Control 2	4,930	93.51	342	6.49
Stroke 1	11,868	84.18	2,230	15.82
Stroke 2	11,274	76.51	3,462	23.49

**Figure 7:**
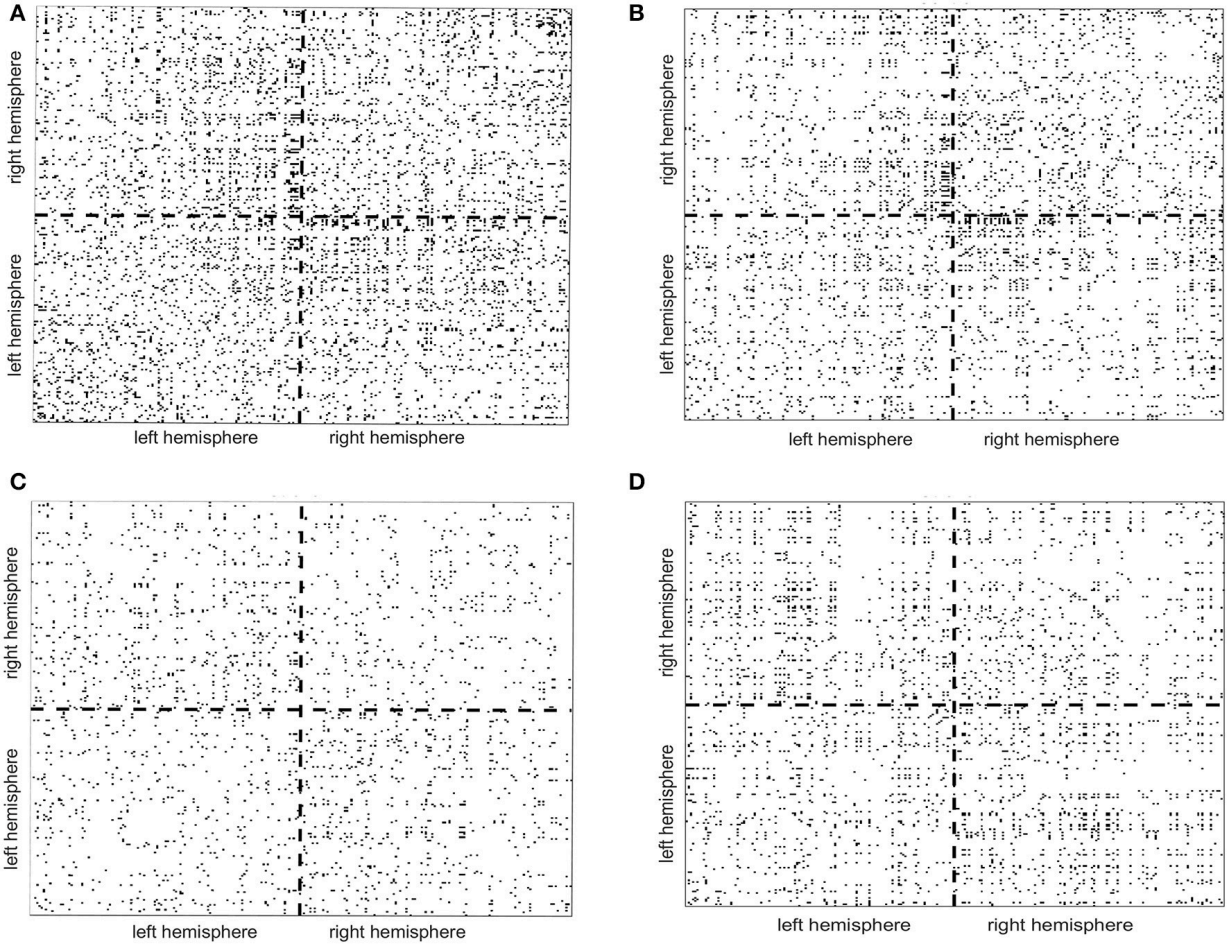
False positives (indicated by the black dots in the maps) of functional connectivity generated by correlation metrics without involving anatomical constraints. **(A,B)** Controls, **(C)** stroke 1, **(D)** stroke 2.

To illustrate how the anatomical priors used in VBMEG improves the estimation of dynamic information flow, we also used a conventional method based on correlation metrics (Greicius et al., 2003) to estimate brain functional connectivity without involving anatomical constraints. As shown in Figure 7, numerous spurious connectivity was estimated between the sources, for which there is no physical pathway connection. It is also quantified in Table 6 as the number of false positives and false discovery rate.

**Table 6:** Number of false positives (FP) and false discovery rate, i.e., FP/(FP + TP) × 100%, generated by correlation metrics without involving anatomical constraints.

	**Number of false positives**	**False discovery rate (%)**
Control 1	5,342	49.05
Control 2	3,598	40.56
Stroke 1	2,084	12.88
Stroke 2	2,896	16.42

*TP, true positive*.

## Discussion

The present work aimed to test the two-stage estimation procedure of the VBMEG method, consisting of an estimation of EEG sources and a dynamic estimation of the information flow between them, in both able-bodied individuals and stroke participants. This study is a proof of principle for the clinical applicability of VBMEG method, not only demonstrating its new application regarding the somatosensory stimulations but also indicating its potential for the study of hemiparetic stroke, which has not been done in previous studies.

The estimation of the activation causality between sources provides insight on functional integration between cortical areas. The selection of strong fiber pairs between estimated sources constrains the solution space. Only the sources having the anatomical connection are included in the estimation of dynamic information flow, which controls the type I error in the MAR modeling. In the VBMEG method, assumptions were made regarding the spatial sparseness and smoothness of the currents, as well as regarding the noise distribution being Gaussian and temporally uncorrelated. However, different noise models can potentially lead to different estimation results, and as long as a “true model” is not known, there will always be uncertainty regarding the possibility of fitting the noise in the solution. Therefore, a quantitative evaluation is needed to assess how much task-relevant cortical source activity and dynamics were captured in the VBMEG method. In this proof of principle study, we assessed the performance of EEG source localization and LCD modeling in the VBMEG method by the VAF (Vlaar et al., 2017; Kalogianni et al., 2018b). The VAF is a summary of how much of the variability of the data can be explained by a fitted model. High VAF for both source localization and LCD modeling was reported in all tested datasets, indicating the VBMEG method can precisely capture the task-relevant cortical source activity and the dynamics in the brain network.

In terms of stroke research, many efforts have been previously made to develop advanced methods based on fMRI to investigate reorganization of the sensorimotor system following a stroke (Grefkes and Fink, 2011). However, the poor temporal resolution of fMRI limits its ability to capture fast somatosensory information flow between cortical regions, which typically occurs in < 100 ms. Therefore, a dynamic method based on EEG is highly desired for studying stroke. Most existing methods computing EEG source interactions are based on signal correlation/coherence (Srinivasan et al., 2007; Smit et al., 2008) or purely signal-driven MAR modeling (Baccalá and Sameshima, 2001; Kaminski et al., 2001; Blinowska et al., 2004; Bressler and Seth, 2011) without referring to anatomical pathways in the brain (Friston, 2011; Sakkalis, 2011). When compared to a conventional method based on correlation metrics (Greicius et al., 2003), it is clear that our method combining the anatomic constraints provided a way to avoid spurious connectivity estimations as shown in Figure 7.

For the able-bodied individuals, the estimated cortical sources and dynamic information flow are found only at the sensorimotor areas contralateral to the finger stimulation. This result is consistent with previous electro-neurophysiological studies (Jamali and Ross, 2013; Porcaro et al., 2013; Kalogianni et al., 2018a), showing that the somatosensory information is processed by brain regions predominantly contralateral to the stimulated hand. Conversely, in chronic hemiparetic stroke participants, the activation of brain activity occurs in both hemispheres, with information flow transmitted from the contralateral (ipsilesional) to the ipsilateral (contralesional) hemisphere in the time period between P50 and N100 whereas in control participants cortical activity stays over the contralateral hemisphere. The result of dynamic information flow indicates that reconfiguration of the sensory network following a stroke. Two chronic stroke survivors have the Fugl-Meyer upper extremity scores of 58 and 66, respectively, and the Erasmus MC modifications to the Nottingham Sensory Assessment (EmNSA) of 8 (see Table 1). Thus, the reconfiguration of the sensory network occurs even in well-recovered individuals with hemiparetic stroke as shown in this study. Similar findings were previously reported in an animal model (Winship and Murphy, 2009). Regarding previous EEG/MRI studies in human participants the focus is on revealing cortical reconfiguration only of a motor network (Ward, 2015). There is growing evidence indicating an increased usage of ipsilateral (contralesional) cortical motor network associated with the loss of independent joint control (van Kordelaar et al., 2012, 2013, 2014) or the expression of the flexion synergy in the paretic upper limb following hemiparetic stroke (Yao et al., 2009; Wilkins et al., 2017; McPherson et al., 2018). However, less is known regarding changes of somatosensory cortical networks in this cohort (Gurari et al., 2017, 2018; Vlaar et al., 2017). Our results could provide new evidence of reconfiguration of somatosensory cortical networks in individuals with hemiparetic stroke, which cannot be revealed by current clinical assessments. The reconfiguration of somatosensory cortical network may contribute to our understanding of time-dependent mechanisms during recovery of the sensory as well as motor function post-hemiparetic stroke (Nelles et al., 1999; Ward, 2017). A better understanding of the recovery of somatosensory function, based on connectivity, is imperative as it serves as an essential feedback channel for the control of movement (Todorov and Jordan, 2002; Scott, 2004). Thus, the VBMEG has potential to evolve into a new neuroimaging tool to monitor cortical network changes post-hemiparetic stroke and thus improving our understanding of stroke recovery.

It is worth to discuss the pros and cons of the presented work, in order to point out possible future directions. This work presented a multi-modal brain imaging method which combines anatomical and physiological information from MRI and EEG. Different from conventional EEG connectivity methods that are purely based on mathematical modeling and signal correlation, our method considers physical connections between cortical sources (obtained from dMRI), which reduces the chance of false positive in connectivity assessment, as indicated by Figure 7 and Table 6. This allows for a comprehensive way to track neural information flow traveling between cortical regions through neural tracts, which, to the best of our knowledge, has never been realized before in other methods. Moreover, compared to the fMRI-based connectivity methods, this EEG-dMRI combined method is able to provide a fine temporal resolution to capture fast somatosensory information flow in the brain, which occurs at the timescale in order of milliseconds.

Nevertheless, the current work has several limitations and could be improved in following directions:

- Ideally, the presented method could be configured in a way that simultaneously estimates EEG sources and dynamic information flow, known as “one-step” strategy (Fukushima et al., 2015). However, the implementation of one-step strategy has yet to be improved and validated^1^. Therefore, in this study, we employed the “two-step” strategy where the EEG source localization and dynamic information were performed sequentially.
- In the future, we will also consider improving our method by estimating tissue conductivity in a subject-specific way. This can be done using the electrical impedance tomography as introduced by Dabek et al. (2016). That will allow a more precise head modeling for EEG source localization.
- Additionally, the white matter conduction velocity could be better estimated in the future by considering the change of fiber myelination after stroke.
- In the current study, we applied our method to stroke patients with small white matter lesions. Thus, the head modeling and EEG source localization would not be affected by the lesion. In the future, the finite element model can be built for precise head modeling, in particular for the patients who also have gray matter lesions. This will also require additional methodological improvements to allow for cortical parcellation. To the best of our knowledge, the currently available approaches are not equipped to solve this problem.
- As a proof of principle study, we did not aim to make general conclusions regarding reorganization of the information flow between neural networks in the brain after stroke. Current results can be considered as a multiple-case study used for the introduction of our methodology as well as provides a preliminary assessment of the ability of our approach. Therefore, no conclusions on a group level can be drawn yet neither for the able-bodied individuals, nor for the stroke survivors. However, we do consider this as an objective for the future application of our method, in order to develop a sensitive biomarker for assessing brain function and reorganization after a hemiparetic stroke. Increase in the inter-hemispheric cross talk after stroke, as indicated by Figure 6 and Table 5, might be considered a candidate for such a biomarker.
- Regarding our results on EEG source localization and information flow, the stimulation to either the left or right hand leads to similar responses in the contralateral hemisphere in able-bodied individuals. Therefore, we did not further investigate the effect of handedness in this study. Furthermore, there are a few previous neuroimaging studies investigated the effects of handedness on the human brain. For example, differences in volumes of gray and white matter areas were detected by Hervé et al. (2006). A voxel-based statistical analysis found higher FA in the left arcuate fasciculus in consistent right-handers (Büchel et al., 2004), but this was not confirmed in a study from Park et al. (2004). Right hand preference might be expected to result from asymmetries in the motor cortex. However, it is more strongly correlated with asymmetries in language-processing structures (Toga and Thompson, 2003). A more recent study by Powell et al. (2012) suggests a greater effect of gender than handedness, based on a DTI analysis. All in all, results regarding handedness effects on the brain have not been entirely consistent across different studies. Based on new evidence from Filatova et al. (2018), it is very likely that the influence of stroke is significantly higher than the effect of handedness. In the future, we would like to further justify this assumption on a larger sample size using our method.

## Conclusion

This study provides a proof-of-principle assessment on the VBMEG method. Our experimental results indicate that VBMEG method can capture the task-relevant cortical source activity and estimate the dynamic information flow in neural networks at the brain. Application of the VBMEG method to the data recorded from stroke participants demonstrates the potential of monitoring dynamic brain activity and revealing the reconfiguration of somatosensory cortical networks following a hemiparetic stroke. In the future, we plan to apply this method to a larger sample size to identify quantitative biomarkers for the assessment of sensory impairment after a unilateral brain injury. Furthermore, the inclusion of this novel imaging method in future clinical trials starting at the acute phase following a hemiparetic stroke is likely to advance our understanding of the neurobiological recovery. In conclusion, the use of the VBMEG method is expected to provide novel quantitative means to assess and subsequently develop more effective neurorehabilitation approaches.

## Author contributions

Conception of the study was conducted by FvdH and YY. All authors participated in design of the study. GK supervised the data acquisition. RT, PM-E, OF, and YY analyzed and interpreted the data with input and support from YT, OY, JD, and FvdH. OF, YY, and JD drafted the manuscript. All authors revised the manuscript critically for important intellectual content. All authors read and approved the manuscript.

### Conflict of interest statement

The authors declare that the research was conducted in the absence of any commercial or financial relationships that could be construed as a potential conflict of interest.

## Footnotes

^1^http://vbmeg.atr.jp/docs/v2/static/vbmeg_users_manual.html#toc9

## Supplementary material

Video 1 Brain information flow for Control 1.

Video 2 Brain information flow for Control 2.

Video 3 Brain information flow for Stroke 1.

Video 4 Brain information flow for Stroke 2.
