# Supplementary material for: Dynamic Information Flow Based on EEG and Diffusion MRI in Stroke: A Proof-of-Principle Study
Source: Front Neural Circuits. 2018 Oct 1;12:79. doi: 10.3389/fncir.2018.00079 (PMC6174251; doi:10.3389/fncir.2018.00079)
Supplement: Supplementary file 1 [file Data_Sheet_1.DOCX]

Supplementary Material

Dynamic information flow based on EEG and diffusion MRI in stroke: a proof-of-principle study

Filatova O.G.*, Yang Y.*, Dewald J.P.A., Tian R., Maceira-Elvira P., Takeda, Y., Kwakkel, G., Yamashita, O., Van der Helm, F.C.T.

*** These authors have contributed equally to this work.**

**Correspondence:**

O. G. Filatova, Email: [O.Filatova@tudelft.nl](mailto:O.Filatova@tudelft.nl) and Y. Yang, Email: [yuan.yang@northwestern.edu](mailto:yuan.yang@northwestern.edu)

# Supplementary Figures

# Please find the detailed overview of the VBMEG method in Supplementary Figure 1


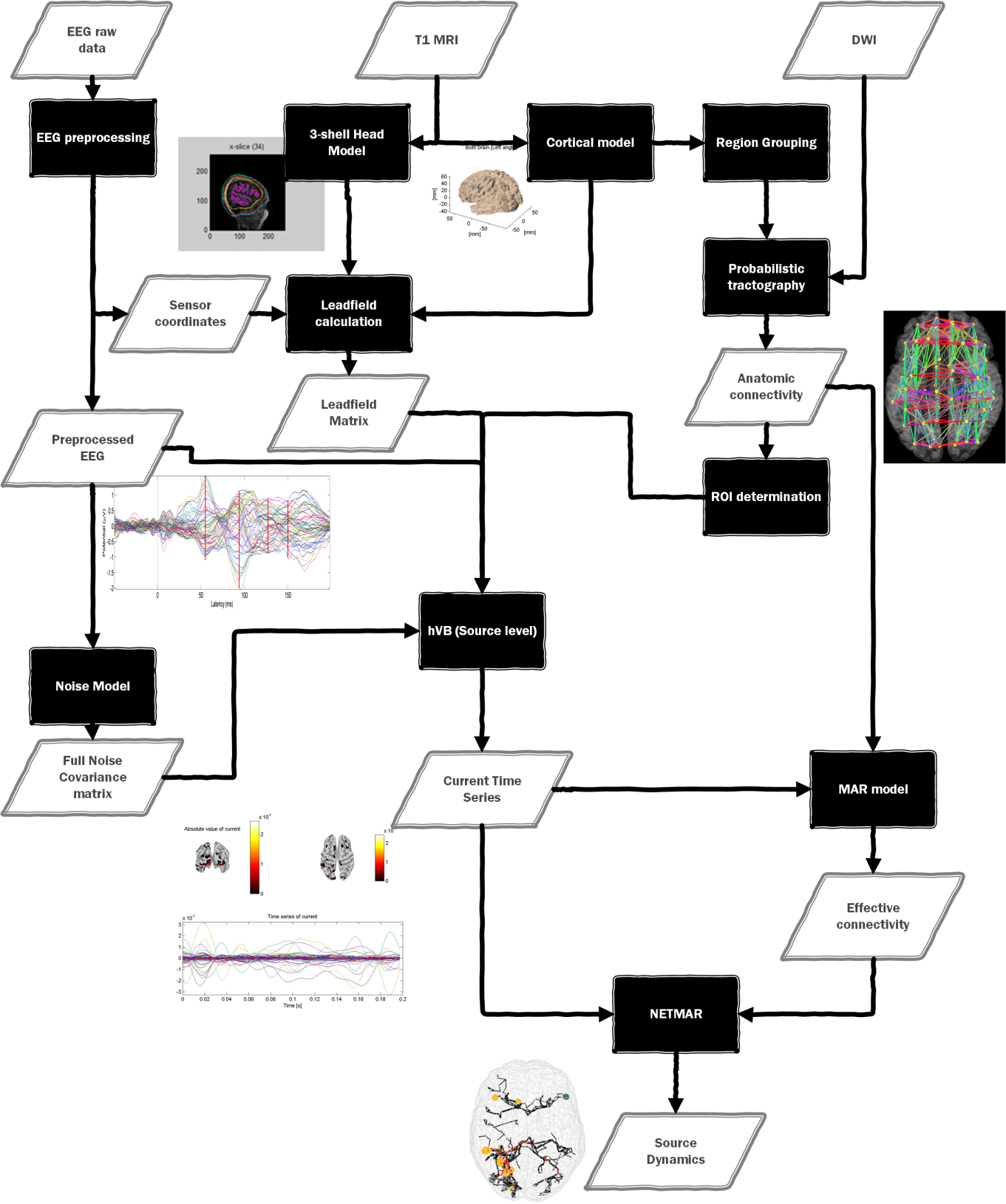


**Supplementary Figure 1.** The detailed overview of the VBMEG method. EEG, T1-weighted MRI and diffusion weighted MRI acquisitions are used as inputs.
